# Mechanism of adipose tissue-derived stromal cell-extracellular vesicles in treating oral submucous fibrosis by blocking the TGF-β1/Smad3 pathway via the miR-760-3p/IGF1R axis

**DOI:** 10.17305/bb.2023.9944

**Published:** 2024-08-01

**Authors:** Fengcong Wang, Li Jiang, Ping Liu, Yanjun Jiang

**Affiliations:** 1 Department of Orthodontics, Jinan Stomatological Hospital, Shandong, China; 2 Department of Endodontics, Jinan Stomatological Hospital, Shandong, China; 3 Engineering Laboratory for Biomaterials and Tissue Regeneration, Ningbo Stomatology Hospital, Zhejiang, China

**Keywords:** Adipose tissue-derived stromal cells (ADSCs), extracellular vesicles (EVs), fibrotic buccal mucosal fibroblasts (fBMFs), insulin-like growth factor 1 receptor (IGF1R), miR-760-3p, oral submucous fibrosis (OSF), TGF-β1/Smad3

## Abstract

Oral submucous fibrosis (OSF) is a prevalent chronic condition, and understanding its pathogenesis is crucial for developing effective therapeutic strategies. This study explores the potential of adipose tissue-derived stromal cell-extracellular vesicles (ADSC-EVs) in mitigating OSF and investigates the underlying molecular mechanisms. OSF was induced in mice by arecoline feeding. Adipose tissue-derived stromal cells (ADSCs), fibrotic buccal mucosal fibroblasts (fBMFs) isolated from OSF mice, and ADSC-EVs were comprehensively characterized. The treatment effects of extracellular vesicles (EVs) and pcDNA3.1-IGF1R on fBMF proliferation, migration, and invasion were assessed using Cell Counting Kit-8 (CCK-8) assay, transwell assay, and flow cytometry assay. The expression levels of alpha-smooth muscle actin (α-SMA), collagen I, collagen III, and insulin-like growth factor 1 receptor (IGF1R) were evaluated by reverse transcription-quantitative polymerase chain reaction (RT-qPCR) and western blot. The interaction between miR-760-3p and IGF1R was investigated. In fBMFs and OSF mice treated with a miR-760-3p inhibitor and/or EVs, the expression patterns of miR-760-3p, IGF1R, and proteins related to the TGF-β1/Smad3 pathway were determined. ADSC-EVs demonstrated the ability to upregulate miR-760-3p, impede cell proliferation, migration, and invasion, and reduce α-SMA, collagen I, and collagen III levels in fBMFs. The expression of miR-760-3p was diminished in ADSC-EVs treated with a miR-760-3p inhibitor. However, silencing miR-760-3p or overexpressing IGF1R partially counteracted the beneficial effects of ADSC-EVs on fBMF fibrosis. miR-760-3p directly targets IGF1R. Significantly, ADSC-EVs exert their suppressive effects on the TGF-β1/Smad3 pathway through the miR-760-3p/IGF1R axis. In summary, ADSC-EVs, by transferring miR-760-3p and inhibiting IGF1R expression, effectively block the TGF-β1/Smad3 pathway, thereby alleviating fibrosis in fBMFs and preventing the progression of OSF.

## Introduction

Oral submucous fibrosis (OSF) is a progressively chronic and precancerous oral lesion with global prevalence. It causes a range of debilitating symptoms, including a burning sensation, intolerance to spicy food, dysphagia, and trismus. OSF is characterized by hyalinization and epithelial atrophy of the oral mucosa, juxta-epithelial inflammation, excessive fibrosis in the lamina propria, and degenerative changes in the muscles [1, 2]. Areca nut chewing, which contains compounds, such as arecoline and arecaidine, along with immunological and genetic factors, as well as nutritional deficiencies, is considered an etiological factor contributing to OSF [3, 4]. The morbidity of OSF varies geographically, with a significant increase observed in the Asian subcontinent, affecting over 5 million people globally across a broad age range [5]. Understanding the mechanisms of OSF and developing effective treatments is critical due to its increasing prevalence and substantial impact on the quality of life. Mesenchymal stromal cells, versatile adult stem cells with the potential for multilineage differentiation, are derived from neuroectoderm and mesoderm [6]. These cells secrete various cytokines and growth factors, playing crucial roles as mediators in the effects of stem cell transplantation. Human exfoliated deciduous teeth stem cells, classified as mesenchymal stem cells due to their origin from the neural crest, exhibit a remarkable capacity for multilineage differentiation and self-renewal. They can differentiate into odontoblasts and are increasingly recognized for their potential in treating periodontal diseases [7]. Adipose tissue-derived stromal cells (ADSCs), a subtype of mesenchymal stromal cells sourced from the adipose tissue, have gained attention for their abundance, reliability, and accessibility [8]. These cells can differentiate into chondroblasts, osteoblasts, and adipocytes. They release extracellular vesicles (EVs) that are crucial for intercellular communication, carrying microRNAs (miRNAs), proteins, and cytokines, thereby playing vital roles in cell signaling and material transfer [9–11]. Stem cell-derived EVs, particularly those from ADSCs (ADSC-EVs), are noted for their stability, low immunogenicity, and manageable dosing, offering potential as therapeutic agents for various diseases [12, 13]. While the significant roles of ADSC-EVs in liver fibrosis have been explored [14], their specific actions in the context of OSF remain less understood.

The diverse biological functions of microRNAs (miRNAs) have established them as crucial regulators in various physiological and pathological processes, offering valuable insights for scientific research and novel avenues for disease treatment [15, 16]. Notably, miR-155 has been identified as a genetic biomarker associated with periodontal parameters and pathogens [17]. While the role of miRNAs in fibrotic conditions like myocardial [18], renal [19], and liver fibrosis [20] has been extensively studied, their exploration in OSF is still emerging. Previous research indicates a downregulation of miR-760 in the buccal mucosa tissues of OSF patients, suggesting its potential role in the disease’s fibrotic etiology [21].

Separately, the insulin-like growth factor 1 receptor (IGF1R), a widely expressed membrane-bound tyrosine kinase, plays a pivotal role in mediating the positive effects of its ligands IGF1 and IGF2, influencing various biological outcomes [22]. The depletion of IGF1R has been associated with impaired endothelial function and increased fibrosis in renal diseases [23]. Interestingly, IGF1R functions through the transforming growth factor (TGF)-β/Smad/STAT cascade, as demonstrated in idiopathic pulmonary fibrosis [24]. TGFs, a family of small secreted signaling proteins, are crucial in regulating essential physiological functions, such as cell proliferation, survival, differentiation, and migration [25]. TGF-β, a member of this family, is involved in a range of pathophysiological processes, including cancer, cardiovascular diseases, skeletal myopathy, and fibrotic lesions [26, 27]. Notably, TGF-β is recognized for its pro-fibrogenic effects, and its excessive activation is considered a key mediator of tissue fibrosis [28]. However, the precise mechanism underlying this process requires further exploration. Therefore, this study delves into the role of ADSC-EVs in OSF by modulating miR-760-3p, aiming to elucidate the potential direction of EV-based treatment for OSF.

## Materials and methods

### Culture and characterization of adipose tissue-derived stromal cells (ADSCs)

Primary ADSCs (ScienCell, Carlsbad, CA, USA) were cultured in a 37 ^∘^C incubator with 5% CO_2_ under saturated humidity. Upon reaching the third passage, flow cytometry was employed to assess the expression of stem cell surface markers (CD45, ab8216; CD31, ab9498; CD105, ab2529; CD90, ab23894; Abcam, Cambridge, UK). The third generation ADSCs underwent culture in adipogenic (Cyagen Biosciences, Santa Clara, CA, USA), osteogenic (Sigma-Aldrich, Merck KGaA, Darmstadt, Germany), and chondrogenic (Sigma-Aldrich) media. The differentiation potential was evaluated using oil red O staining (Sigma-Aldrich), Alizarin red staining (Cyagen Biosciences), and Alcian blue glacial acetic acid staining (REGAL, Shanghai, China) [29, 30].

### Extraction and identification of extracellular vesicles (EVs)

ADSCs were cultured for 48 h in Dulbecco's modified eagle's medium (DMEM) or RPMI-1640 medium supplemented with 10% EVs-depleted fetal bovine serum (FBS) (Thermo Fisher Scientific, Waltham, MA, USA) and 1% penicillin–streptomycin. The collected supernatant underwent centrifugation at 300×*g* for 5 min to eliminate cells and cell debris, followed by centrifugation at 2000×*g* for 30 min to remove larger vesicles. The supernatant was then filtered with a 0.2-µm filter membrane (Millipore, Billerica, MA, USA), followed by centrifugation at 100,000×*g* for 2 h. EV particles were washed with phosphate-buffered saline (PBS) and centrifuged at 100,000×*g* for 2 h, followed by resuspension in PBS. Similarly, ADSCs were cultured in EVs-depleted FBS medium with the addition of GW4869 (exosome inhibitor, 20-µg/mL conditioned medium; Sigma-Aldrich) for 48 h, with the conditioned medium serving as the control (GW4869 group). The expression of EV surface markers (CD9, ab92726; CD63, ab134045; Calnexin, ab22595; Abcam) was assessed via western blot. The morphology of EVs was visualized using a transmission electron microscope (TEM), and the particle size of EVs was determined through nanoparticle tracking analysis (NTA) using NanoSight NS300 (Malvern Panalytical, Kassel, Germany).

### Animal grouping and treatment

BALB/c male mice (4 weeks old, weighing 20–22 g) were obtained from Pengyue Experimental Animal Breeding Co. [SCXK (Shandong) 2022-0006, Jinan, Shandong, China]. Mice were housed under a 24-h alternating light cycle with 30%–50% relative humidity and provided standard mouse fodder. Arecoline (Sigma-Aldrich) was diluted to 1000 mg/L in distilled water.

The mice were categorized as follows (*N* ═ 12): 1) Control group: fed with distilled water without any addition; 2) OSF group: fed with an isodose arecoline solution; 3) OSF + EVs group: fed with the arecoline solution and injected with 100 µL PBS solution containing 1 × 10^10^ particles of EVs every three days via tail vein [31]; 4) OSF + EVs-miR-inhi group: fed with the arecoline solution and injected with an equal amount of PBS solution containing 1 × 10^10^ particles of EVs-miR-730-3p-inhibitor every three days via tail vein; and 5) OSF + EVs-miR-NC group: fed with the arecoline solution and injected with an equal volume of PBS solution containing 1 × 10^10^ particles of EVs-miR-730-3p-negative control (NC) every three days via tail vein. The water was changed weekly. After 21 days of ad libitum feeding, mice were euthanized with intraperitoneal administration of 800 mg/kg/d pentobarbital sodium. In each group, buccal tissues from six mice were prepared into paraffin sections for hematoxylin–eosin (HE), Masson staining, and immunohistochemistry. Another portion of buccal tissues was ground in liquid nitrogen to form a tissue homogenate for subsequent reverse transcription-quantitative polymerase chain reaction (RT-qPCR) and western blot assays. Tissues from another six mice were used to isolate and extract cells for experiments.

### Staining of oral mucosal lesion specimens

After euthanasia, mice were fixed in a supine position. Buccal mucosa tissues were removed under sterile conditions, fixed with 10% formalin, embedded in paraffin, and cut into 4-mm sections. HE and Masson staining were performed to detect histopathological changes in oral submucosa.

### Isolation and culture of mouse fibroblasts

Mouse buccal tissues were rinsed thrice with cold PBS and soaked for 5 min with biclonal antibody and amphotericin. The tissues were then cut into small pieces (approximately 1 mm^3^), placed in culture bottles, and incubated for 10 min. A small amount of DMEM medium containing 20% neonatal calf serum was added, and the medium was replaced with fresh medium after three days. Adherent cells from the second generation were detached with 0.25% trypsin for 1 min, and the reaction was stopped by adding 20% neonatal calf serum. The suspended cells were adjusted to 2 × 10^6^/mL and then placed in 6-well plates containing sterile slides.

### Characterization of fibrotic buccal mucosal fibroblasts (fBMFs)

Cells were cultured for 3–5 days and characterized by immunohistochemical staining. The second generation adherent cells were detached with 0.25% trypsin, resuspended in DMEM containing 10% FBS (adjusted to a density of 2 × 10^6^ cells/mL), and dripped onto 6-well plates with sterile slides. The cell culture fluid was discarded after more than 80% of the cells adhered to the wall. Slides were removed, rinsed with PBS, fixed with 4% paraformaldehyde for approximately 10–15 min, and washed three times with PBS. The slides were then incubated for 10 min with 0.5% Triton X-100, rinsed thrice with PBS, incubated with 3% H_2_O_2_ for 5 min, and again rinsed three times with PBS. After blocking with goat serum for 10 min, slides were incubated overnight with primary antibodies (Pan-CK: 1:400; vimentin: 1:200; Boster, Wuhan, Hubei, China) at 4 ^∘^C. Slides were then placed in a 37 ^∘^C incubator for 30 min, rinsed thrice with PBS, and incubated for 15 min with a secondary antibody (1:200, ZSGB-BIO, Beijing, China) at 37 ^∘^C. Subsequently, slides were developed with diaminobenzidine (ZSGB-BIO) for 2 min, stained with hematoxylin (#14166, CST, San Antonio, TX, USA), mounted with neutral resin, and observed under an optical microscope (Olympus, Tokyo, Japan).

### EVs uptake assay

EV particles were resuspended in 1-mL diluent C following the manufacturer’s instructions. In addition, 2 µL of PKH-26 (Sigma-Aldrich) was mixed with 245 µL of diluent C. EV suspension was co-incubated with a staining solution for 5 min, and an equal volume of 1% bovine serum albumin was added. fBMFs were then seeded in 24-well plates, and PKH-26 dye-labeled EVs (10 µg) were added to the medium. After 4 h, cells were fixed with 4% formaldehyde, and nuclei were stained with 4’,6-diamidino-2-phenylindole (DAPI) (Sigma-Aldrich). The uptake of EVs by fBMFs was observed under a laser scanning confocal microscope (LSM710, Zeiss, Oberkochen, Germany).

### RNase protection assay

RNase treatment was performed to determine whether miR-760-3p was encapsulated in EVs or bound to the surface of EVs. EVs were resuspended in PBS and incubated with 2 µg/µL RNase (Purelink RNase technologies, Thermo Fisher Scientific) for 20 min at 37 ^∘^C. Subsequently, EVs were treated with 0.1% TritonX 100 for 20 min to disrupt the membrane integrity of EVs, followed by incubation with RNase A. This reaction was terminated by adding lysis buffer, and RNA was isolated to determine miR-760-3p expression.

### Cell transfection

miR-760-3p inhibitor, pcDNA3.1-IGF1R, and their corresponding NCs were provided by RiboBio (Guangzhou, Guangdong, China). ADSCs at the exponential phase were seeded in 6-well plates at 1 × 10^5^ cells/well. Upon reaching 70% confluence, ADSCs or fBMFs were transfected for 48 h using Lipofectamine 2000 (Invitrogen, Carlsbad, CA, USA).

### RT-qPCR

Total RNA extraction from cells was performed using the TRIzol reagent (Invitrogen), followed by cDNA synthesis using PrimeScript RT reagent kits (Takara, Dalian, Liaoning, China). qPCR was performed with SYBR^®^ Premix Ex Taq^TM^ II (Takara) on an ABI7900HT fast PCR real-time system (ABI, Foster City, CA, USA). The thermal cycling conditions included pre-denaturation at 95 ^∘^C for 10 min, followed by 40 cycles of denaturation at 95 ^∘^C for 10 s, annealing at 60 ^∘^C for 20 s, and extension at 72 ^∘^C for 34 s. GAPDH and U6 were internal references, and data were analyzed using the 2^-ΔΔCt^ method [32]. Primer sequences are shown in Table 1.

**Table 1:** Primer sequences

**Gene**	**Forward 5’-3’**	**Reverse 5’-3’**
miR-760-3p	GGCTCTGGGTCTGTGGG	GAACATGTCTGCGTATCTC
*IGF1R*	GACCTCTTCCCGAACCTC	TGTAGTTATTGGACACCGCAT
*U6*	CGCTTCGGCAGCACATATAC	AATATGGAACGCTTCACGA
*GAPDH*	ATGGTTTACATGTTCCAATATGA	TTACTCCTTGGAGGCCATGTGG

IGF1R: Insulin-like growth factor 1 receptor; GAPDH: Glyceraldehyde-3-phosphate dehydrogenase.

### Dual-luciferase reporter assay

Binding sites between miR-760-3p and IGF1R were predicted using StarBase (https://starbase.sysu.edu.cn/), Jefferson (https://cm.jefferson.edu/), and miRDB (http://mirdb.org/) databases. The IGF1R-wild-type (WT) plasmid and corresponding IGF1R-mutant (MUT) plasmid were constructed by amplifying complementary binding sequences and mutation sequences of miR-760-3p and IGF1R and cloning the sequences into pmiR-GLO luciferase vectors (Promega, Madison, WI, USA). The plasmids were co-transfected into fBMFs with mimics NC or miR-760-3p mimics (GenePharma, Shanghai, China) using Lipofectamine^TM^ 2000. Luciferase activity was evaluated after 48-h transfection.

### Western blot analysis

Cells were lysed using an enhanced radio-immunoprecipitation assay solution containing protease inhibitor (Beyotime, Shanghai, China) to obtain protein samples. Protein concentration in the supernatant was measured using bicinchoninic acid kits (Beyotime). Proteins were separated by 10% sodium dodecyl sulfate-polyacrylamide gel electrophoresis and electrotransferred onto polyvinylidene fluoride (PVDF) membranes (Millipore). PVDF membranes were subsequently blocked with 5% skim milk-Tris-buffered saline-tween (TBST) for 1–2 h. Membranes were probed overnight with primary antibodies, including anti-GAPDH (1:10,000, ab181602, Abcam), anti-IGF1R (1:1000, ab182408 Abcam), anti-collagen I (1:1000, ab260043, Abcam), anti-collagen III (1:1000, ab7778, Abcam), anti-α-smooth muscle actin (α-SMA) (1:10,000, ab124964, Abcam), anti-TGF-β1 (1:1000, ab215715, Abcam), anti-p-Smad3 (1:1000, ab63402, Abcam), and anti-Smad3 (1:1000, ab40854, Abcam) at 4 ^∘^C. After rinsing with TBST thrice for 10 min each, membranes were incubated with horseradish peroxidase-labeled goat anti-rabbit IgG (1:5000, CoWin Biosciences, Beijing, China) for 1 h and then rinsed thrice with TBST for 10 min each. Bands were visualized using chemiluminescence, and grayscale analysis was conducted with GAPDH as an internal control.

### Cell Counting Kit-8 (CCK-8) assay

Cells were seeded in 96-well plates at 2 × 10^4^ cells/mL. After 24 h, cell viability was assessed with a CCK-8 assay. Each well was supplemented with the corresponding volume of CCK-8 reagent, mixed, and cultured in a 37 ^∘^C incubator with 5% CO_2_ for another 1.5 h. The optical density (OD) value at 450 nm was then determined on a microplate reader.

### Flow cytometry

Cells were plated in 12-well plates at a density of 2 × 10^5^ cells/mL, and apoptosis was assessed using Annexin V Dead Cell kits (Beyotime) on a flow cytometer (Beckman Coulter, Brea, CA, USA).

### Transwell assays

Migration and invasion analyses were conducted using a 24-well plate Transwell^®^ system (Corning, Corning, NY, USA) with a polycarbonate membrane (8-µm pore size). For invasive ability assessment, membranes were coated with Matrigel. Cells (1 × 10^5^) were added to the apical chamber in 250 µL serum-free medium, and the basolateral chamber was filled with 10% FBS. After 48 h of incubation, the filter membranes were stained with 0.1% crystal violet. Subsequently, cells were observed under an inverted microscope (×100), and counts were performed in five different fields.

### Ethical statement

This study received approval from the Ethics Committee of Ningbo Stomatology Hospital (approval number: JNSKQYY-2022-021), and conscious efforts were made to minimize the number of animals used and mitigate any potential pain.

### Statistical analysis

Data analysis was performed using SPSS 21.0 (IBM Corp., Armonk, NY, USA) software, and GraphPad Prism 8 software was adopted for plotting. The data, confirmed to have a normal distribution via the Kolmogorov–Smirnov test, are presented as mean ± standard deviation (SD). The independent sample *t*-test was employed for comparisons between two groups, and one-way analysis of variance (ANOVA) was conducted for comparisons among multiple groups, followed by Tukey’s test. The *P* value was obtained from a two-sided test, and *P* < 0.05 was considered statistically significant.

## Results

### Extraction and characterization of ADSCs and EVs

**Figure 1.**
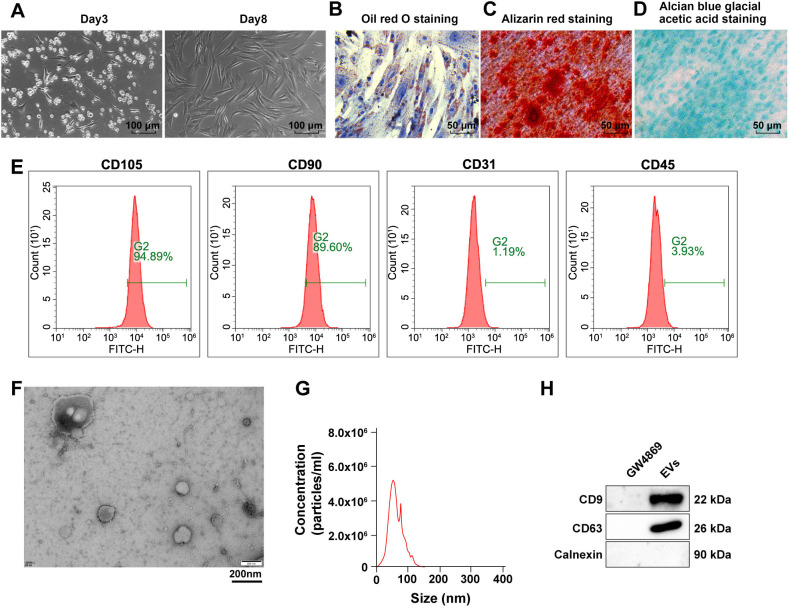
**Extraction and characterization of ADSCs and ADSC-EVs.** (A) Morphological changes in ADSCs observed under a microscope; (B–D) Adipogenic, osteogenic, and chondrogenic abilities of ADSCs assessed by oil red O staining, Alizarin red staining, and Alcian blue glacial acetic acid staining, respectively; (E) ADSC surface markers CD105, CD90, CD45, and CD31 detected by flow cytometry; (F) Morphology observed under TEM; (G) Particle size of EVs measured using NTA; (H) Surface markers of EVs detected by western blot. ADSC: Adipose tissue-derived stromal cell; EV: Extracellular vesicle; TEM: Transmission electron microscope; NTA: Nanoparticle tracking analysis.

Initially, the majority of ADSCs displayed a long shuttle-shaped morphology on days 3 and 8 of culture, as illustrated in Figure 1A. Adipogenic induction on day 14 revealed red staining with oil red O (Figure 1B), indicating the presence of fat droplets in ADSCs. Osteogenic induction on day 21 resulted in Alizarin red-stained calcium precipitates surrounding the cells, as shown in Figure 1C. Following chondrogenic induction on day 14, the cells exhibited typical chondrocyte morphology with blue-stained precipitates, as depicted in Figure 1D. These results collectively confirmed the osteogenic, adipogenic, and chondrogenic differentiation capabilities of ADSCs. Flow cytometry analysis further validated this by confirming the positive expression of CD105 (94.89%) and CD90 (89.6%), and the negative expression of CD31 (1.19%) and CD45 (3.93%), as seen in Figure 1E. This confirmed the successful culture of ADSCs. Subsequently, ADSC-EVs were isolated and characterized. Transmission electron microscopy (TEM) revealed that these vesicles had round, cup-shaped structures with a uniform size, a clear bilayer lipid membrane, and a diameter ranging approximately from 40 to 100 nm (Figure 1F). NTA showed a size distribution primarily between 40 and 100 nm, with a peak diameter of around 55 nm (Figure 1G). Western blot analysis demonstrated significant expression of CD9 and CD63 in ADSC-EVs, while Calnexin was not expressed (Figure 1H). These results collectively confirmed the successful extraction and characterization of ADSC-EVs.

**Figure 2.**
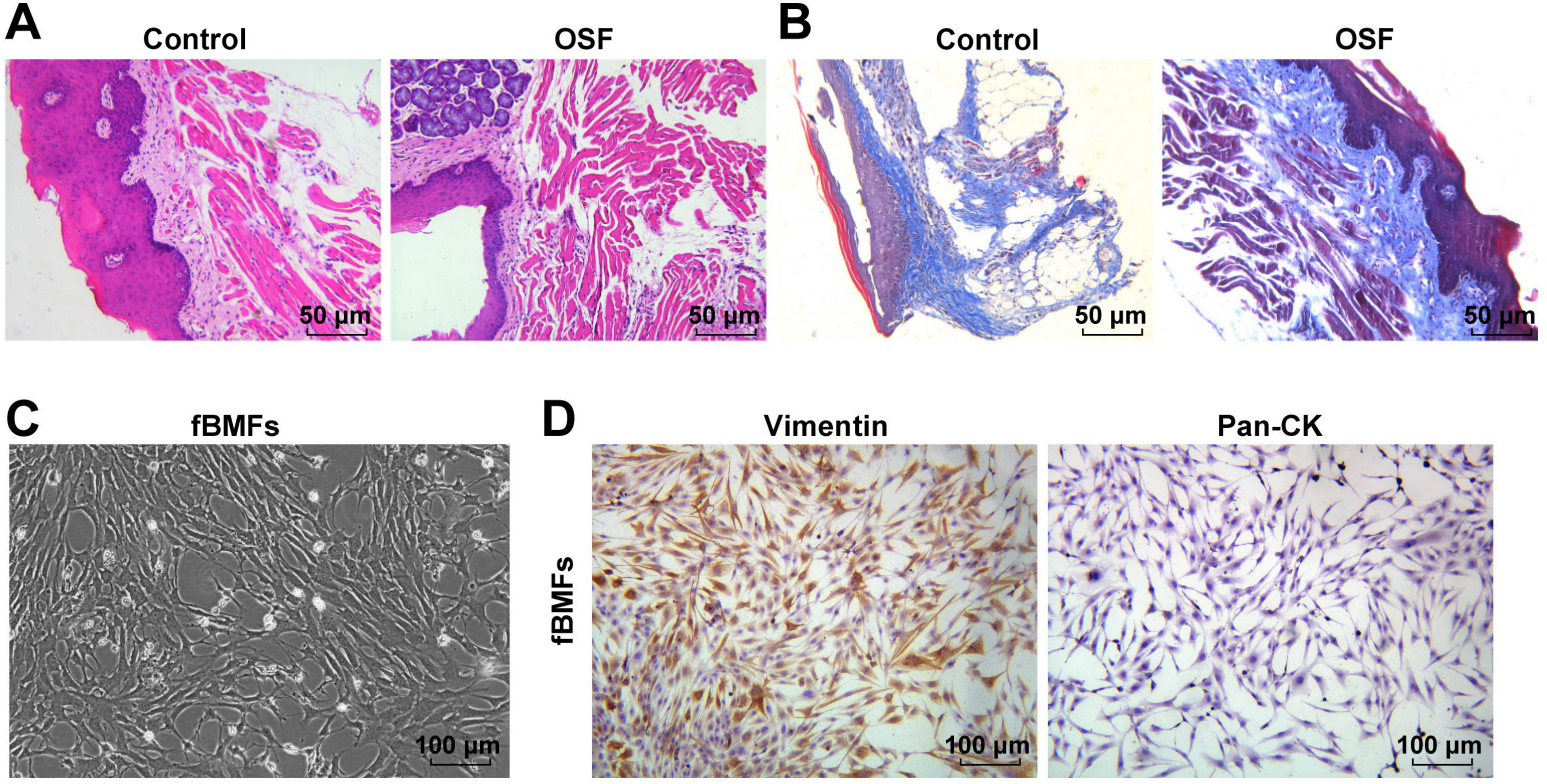
**Establishment of arecoline-induced OSF mouse models.** (A and B) Pathological changes in oral submucosa tissues assessed by HE and Masson staining; (C) Morphology of fBMFs observed under a microscope; (D) Expression levels of vimentin and keratin detected by immunohistochemistry. OSF: Oral submucous fibrosis; HE: Hematoxylin–eosin; fBMFs: Fibrotic buccal mucosal fibroblasts.

### Establishment of arecoline-induced oral submucous fibrosis mouse models

After 21 days of feeding mice with arecoline solution, collected oral tissues exhibited characteristic features of OSF. HE staining revealed epithelial atrophy of buccal mucosa, gradual disappearance or thinning of epithelial papillae, thickening of the lamina propria, infiltration of inflammatory cells, and reduced lamina propria vessels in the OSF group compared to the control group (Figure 2A). Masson staining indicated increased blue-violet stained collagen in the OSF group, primarily distributed in the lamina propria of buccal mucosa (Figure 2B). Subsequently, fBMFs were successfully extracted and characterized, presenting shuttle- and polygon-shaped morphology under the microscope (Figure 2C). Immunohistochemical results showed a positive expression of vimentin and a negative expression of keratin (Pan-CK), with shuttle- and polygon-shaped cell morphology, blue-stained nuclei, brownish-yellow cytoplasm, and clear nuclear membranes (Figure 2D). In conclusion, arecoline successfully induced OSF in mice, and fBMFs were effectively separated.

### ADSC-EVs alleviated fibrosis in fBMFs by transporting miR-760-3p in vitro

**Figure 3.**
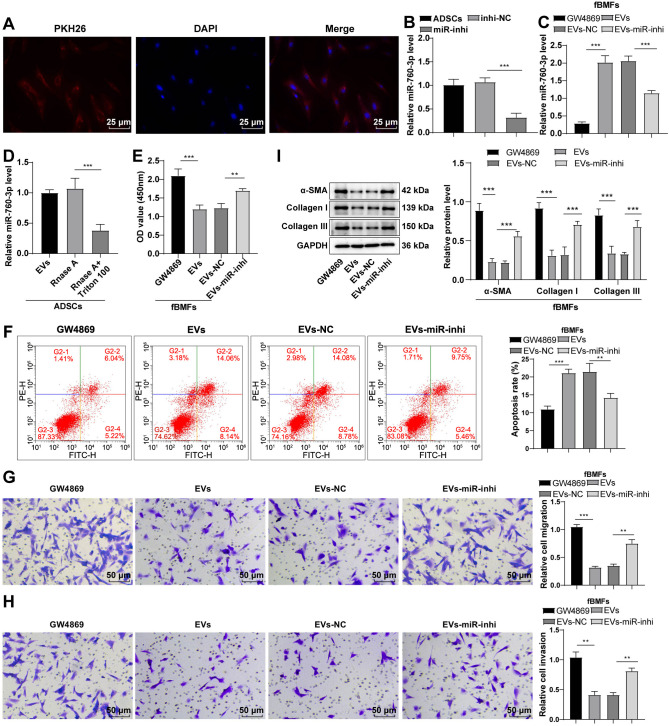
**ADSC-EVs ameliorated the fibrosis of fBMFs by carrying miR-760-3p in vitro.** The cell experiment was performed three times, and data are presented as mean ± standard deviation. One-way ANOVA was used for comparisons among multiple groups, followed by Tukey’s test. **P < 0.01, ***P < 0.001. (A) Uptake of EVs by fBMFs; nuclei of fBMFs were labeled with DAPI (blue), and EVs were labeled with PKH26 (red); (B and D) RT-qPCR determined miR-760-3p expression; (E) CCK-8 assay assessed the viability of fBMFs; (F) Flow cytometry assessed the apoptosis of fBMFs; (G and H) Transwell assay assessed migration and invasion of fBMFs; (I) Western blot determined the expression of fibrosis marker proteins α-SMA, collagen I, and collagen III. ADSC: Adipose tissue-derived stromal cell; EV: Extracellular vesicle; fBMFs: Fibrotic buccal mucosal fibroblasts; ANOVA: Analysis of variance; DAPI: 4’,6-Diamidino-2-phenylindole; RT-qPCR: Reverse transcription-quantitative polymerase chain reaction; CCK-8: Cell Counting Kit-8; α-SMA: Alpha-smooth muscle actin; miRNA: microRNA.

Following a 24-h co-incubation of fBMFs with ADSC-EVs, the uptake assay demonstrated the gradual internalization of PKH26-labeled EVs (red dots) by fBMFs (Figure 3A), indicating effective endocytosis. Given the low expression of miR-760 in OSF patients [21] and the high expression in blood EVs (http://bioinfo.life.hust.edu.cn/EVmiRNA/#!/), we hypothesized that ADSC-EVs might influence fBMF fibrosis by transporting miR-760. To test this, a miR-760-3p inhibitor was introduced into ADSCs, resulting in suppressed miR-760-3p expression (*P* < 0.001) (Figure 3B). EVs isolated from these cells were then used to treat fBMFs, leading to a reduction in miR-760-3p expression of miR-760-3p in fBMFs compared to the EVs-NC group (*P* < 0.001) (Figure 3C). The RNase assay further confirmed that miR-760-3p was encapsulated in EVs (Figure 3D).

Subsequent experiments explored whether ADSC-EVs influenced the fibrosis progression of fBMFs by carrying miR-760-3p. Culturing fBMFs with EVs from different treatment groups revealed that EVs significantly inhibited fBMF viability (*P* < 0.01) (Figure 3E). Flow cytometry demonstrated that ADSC-EVs promoted fBMF apoptosis (Figure 3F). Transwell assay revealed that ADSC-EVs markedly reduced fBMF migration and invasion (all *P* < 0.01) (Figure 3G and 3H). Western blot analysis showed that EV treatment significantly suppressed the levels of fibrosis marker proteins α-SMA, collagen I, and collagen III (all *P* < 0.001) (Figure 3I). However, the knockdown of miR-760-3p in EVs partially reversed these effects (all *P* < 0.01) (Figure 3E–3I). Altogether, ADSC-EVs mitigated the fibrosis of fBMFs by transporting miR-760-3p in vitro.

### miR-760-3p targeted IGF1R

**Figure 4.**
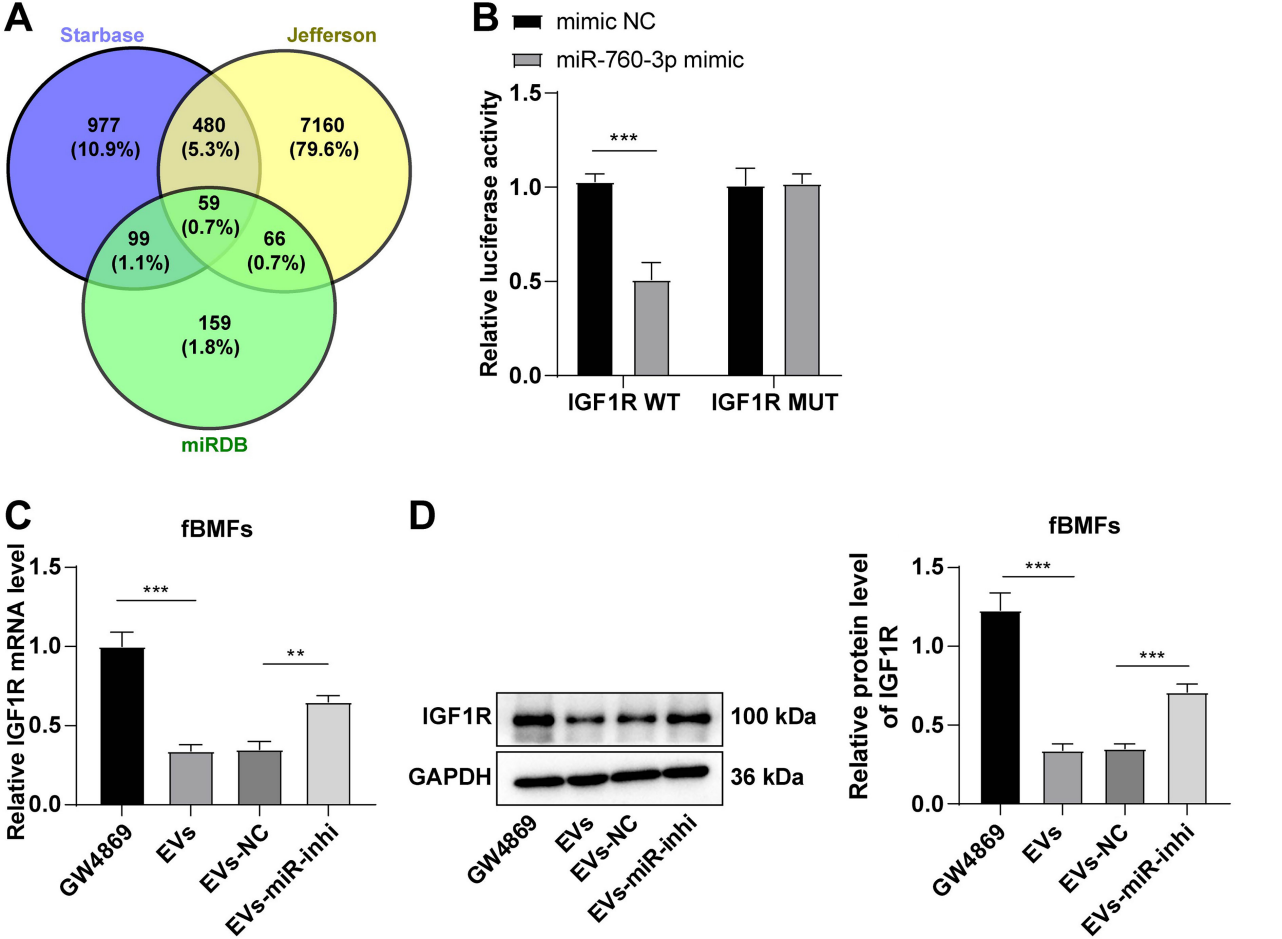
**miR-760-3p targeted IGF1R expression.** The cell experiment was performed three times, and data are presented as mean ± standard deviation. The independent sample *t*-test was used for comparisons between two groups in panel B, and one-way ANOVA was used for comparisons among multiple groups in panels C and D, followed by Tukey’s test. **P < 0.01, ***P < 0.001. (A) Venn diagram of target genes of miR-760-3p predicted by StarBase, Jefferson, and miRDB databases; (B) Dual-luciferase assay verified the targeted binding between miR-760-3p and IGF1R; (C and D) RT-qPCR and western blot determined IGF1R mRNA and protein expressions. ANOVA: Analysis of variance; IGF1R: Insulin-like growth factor 1 receptor; RT-qPCR: Reverse transcription-quantitative polymerase chain reaction; NC: Negative control.

Building on the demonstrated impact of ADSC-derived miR-760-3p on fBMF fibrosis, downstream target genes were investigated. The StarBase, Jefferson, and miRDB databases identified potential target genes, with the IGF1R emerging as a candidate (Figure 4A). IGF1R, a membrane-bound tyrosine kinase, has known implications in fibrotic lung disease pathogenesis [33, 34]. The dual-luciferase assay validated the targeted binding between miR-760-3p and IGF1R, evidenced by significantly reduced luciferase activity in cells co-transfected with miR-760-3p mimics and IGF1R-WT plasmids (*P* < 0.001). However, no significant change was observed in cells co-transfected with miR-760-3p mimics and IGF1R-MUT plasmids (*P* > 0.05) (Figure 4B).

Further examination of IGF1R expression in fBMFs treated with differently processed EVs revealed that EVs significantly reduced IGF1R expression, while the inhibition of miR-760-3p expression in EVs increased IGF1R expression (all *P* < 0.01) (Figure 4C and 4D). In summary, miR-760-3p directly targeted IGF1R, leading to the repression of IGF1R expression.

### IGF1R overexpression partially counteracted the anti-fibrotic impact of ADSC-EVs on fBMFs

**Figure 5.**
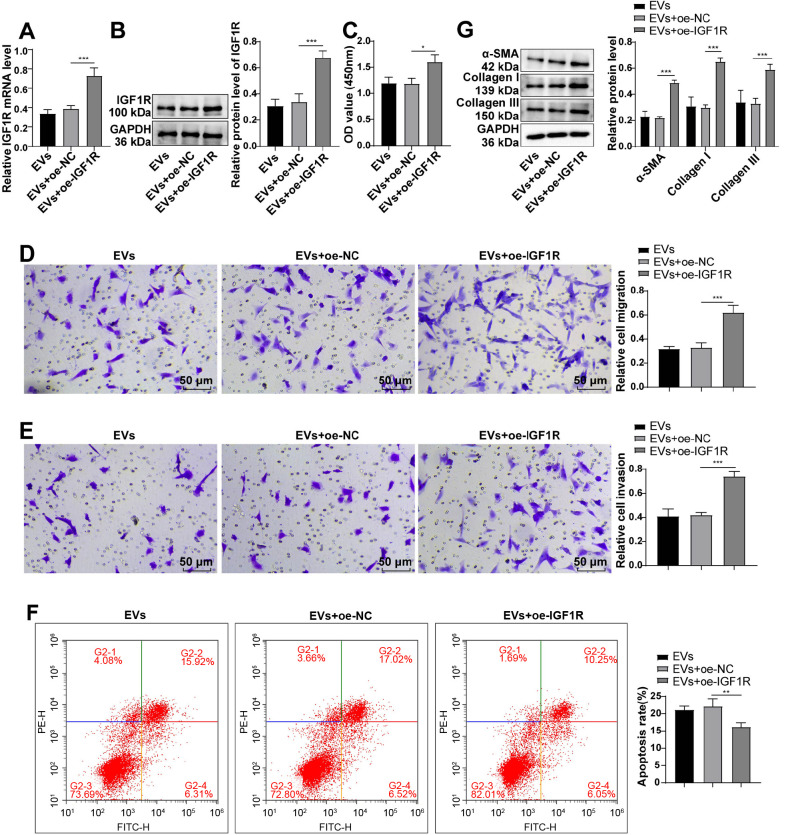
**Overexpression of** **IGF1R partially reversed the alleviating effect of** **ADSC-EVs on fibrosis of** **fBMFs.** The cell experiment was performed three times and data are presented as mean ± standard deviation. One-way ANOVA was conducted for comparisons among multiple groups, followed by Tukey’s test. *P < 0.05, **P < 0.01, ***P < 0.001. (A and B) RT-qPCR and western blot detected the expression of IGF1R; (C) CCK-8 assay evaluated cell proliferation; (D and E) Transwell assays evaluated cell migration and invasion; (F) Flow cytometry evaluated cell apoptosis; (G) Western blot determined the expression levels of α-SMA, collagen I, and collagen III. ANOVA: Analysis of variance; IGF1R: Insulin-like growth factor 1 receptor; ADSC: Adipose tissue-derived stromal cell; EV: Extracellular vesicle; fBMFs: Fibrotic buccal mucosal fibroblasts; RT-qPCR: Reverse transcription-quantitative polymerase chain reaction; CCK-8: Cell Counting Kit-8; α-SMA: Alpha-smooth muscle actin.

To assess the involvement of IGF1R in the observed effects, we induced IGF1R overexpression in fBMFs during EV treatment. RT-qPCR and western blot analyses confirmed a substantial increase in IGF1R expression in fBMFs (all *P* < 0.001) (Figure 5A and 5B). Notably, IGF1R overexpression reversed the inhibitory effects of EVs on fBMF proliferation, migration, and invasion (all *P* < 0.05) (Figure 5C–5F). Additionally, the elevated expression of α-SMA, collagen I, and collagen III further confirmed the reversal of anti-fibrotic effects by IGF1R overexpression (all *P* < 0.001) (Figure 5G). In summary, ADSC-EVs exerted their anti-fibrotic effects on fBMFs by suppressing IGF1R.

### ADSC-EVs attenuated fBMF fibrosis by inhibiting the TGF-β1/Smad3 pathway via IGF1R

**Figure 6.**
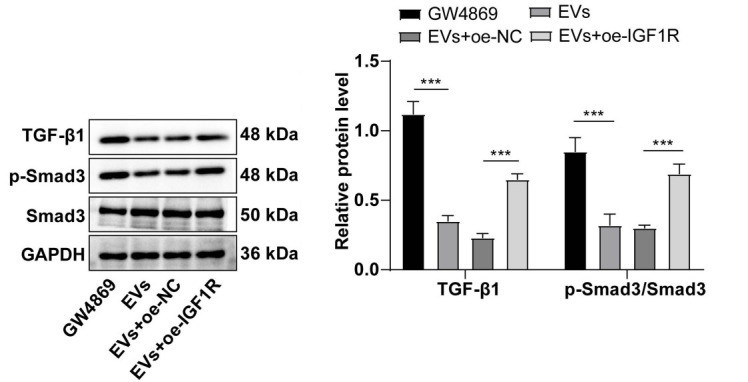
**ADSC-EVs alleviating the fibrosis of fBMFs via repressing the activation of the TGF-β1/Smad3 pathway through IGF1R.** The cell experiment was performed three times, and data are presented as mean ± standard deviation. One-way ANOVA was used for comparisons among multiple groups, followed by Tukey’s test. ***P < 0.001. Western blot measured the levels of TGF-β1/Smad3 pathway-associated proteins. ADSC: Adipose tissue-derived stromal cell; EV: Extracellular vesicle; fBMFs: Fibrotic buccal mucosal fibroblasts; ANOVA: Analysis of variance; NC: Negative control.

Recognizing the crucial role of the TGF-β1/Smad3 pathway in fibrosis [35], we explored the potential influence of IGF1R on this pathway, western blot results demonstrated that the addition of ADSC-EVs significantly reduced TGF-β1, p-Smad3, and Smad3 levels in fBMFs. However, the subsequent overexpression of IGF1R markedly elevated these levels (all *P* < 0.001) (Figure 6). Overall, ADSC-EVs ameliorate fBMF fibrosis by suppressing the activation of the TGF-β1/Smad3 pathway through IGF1R.

**Figure 7.**
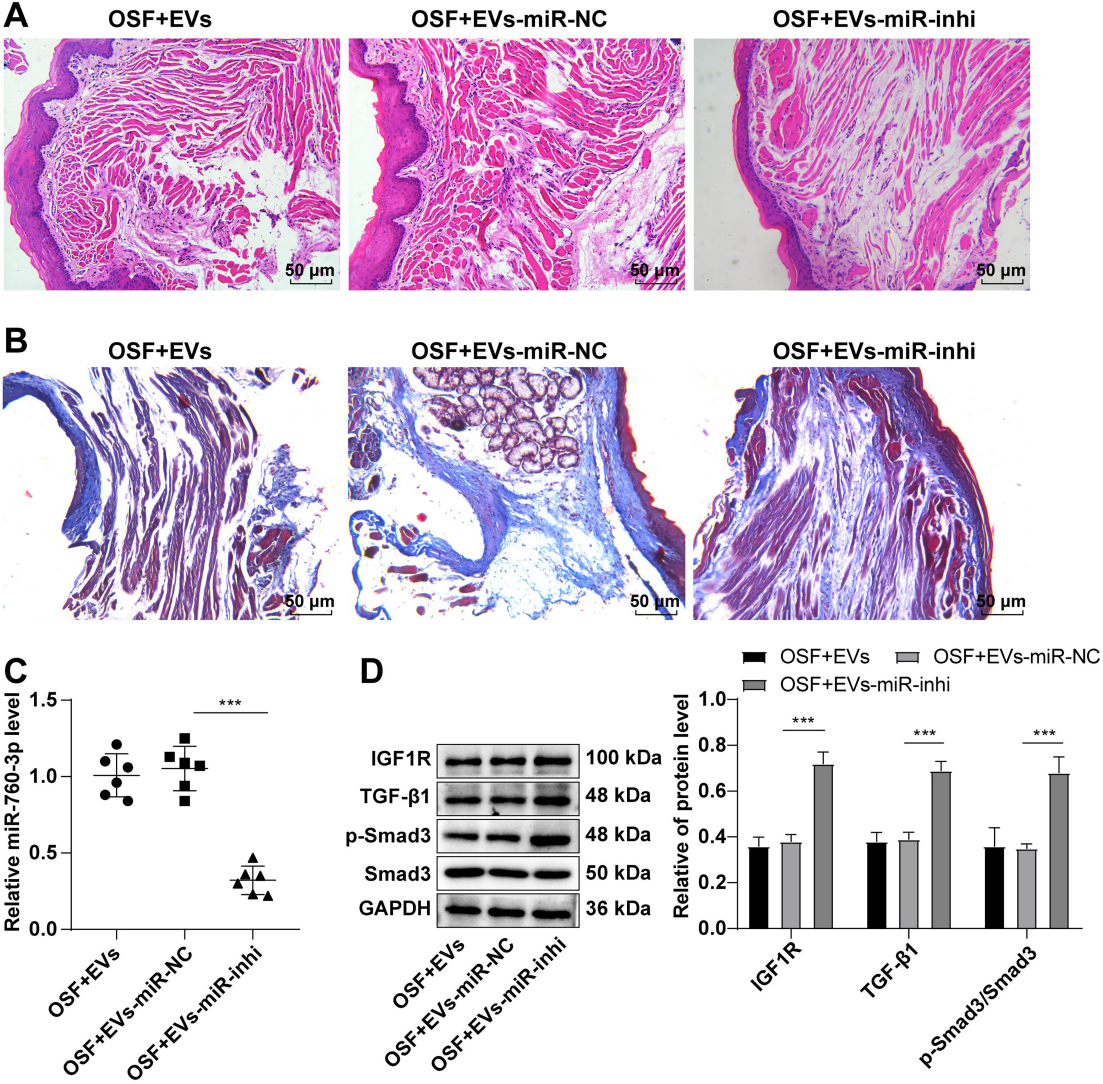
**ADSC-EVs ameliorated OSF by inhibiting the TGF-β1/Smad3 pathway via mediating the miR-760-3p/IGF1R axis.** *N* ═ 6, data are presented as mean ± standard deviation, one-way ANOVA was employed for comparisons among multiple groups, followed by Tukey’s test. ***P < 0.001. (A and B) Pathological changes in oral submucosa tissues assessed by HE staining and Masson staining; (C) RT-qPCR detected miR-760-3p levels; (D) Western blot determined the expression levels of IGF1R and TGF-β1/Smad3 pathway-related proteins. ADSC: Adipose tissue-derived stromal cell; EV: Extracellular vesicle; IGF1R: Insulin-like growth factor 1 receptor; HE: Hematoxylin–eosin; RT-qPCR: Reverse transcription-quantitative polymerase chain reaction; OSF: Oral submucous fibrosis; NC: Negative control.

### ADSC-EVs alleviated OSF by modulating miR-760-3p/IGF1R in mice

To ascertain whether ADSC-EVs impact OSF through the miR-760-3p/IGF1R axis, OSF mouse models were injected with EVs and EVs-miR-730-3p-inhibitor via the tail vein. HE staining revealed that miR-760-3p inhibition in EVs resulted in mouse buccal mucosal epithelium contraction, increased inflammatory cell infiltration, and decreased blood vessels in the lamina propria compared to EVs-treated OSF mice (Figure 7A). Masson staining demonstrated enhanced blue-purple stained collagen in OSF mice treated with EVs-miR-730-3p-inhibitor (Figure 7B). Relative to the OSF + EVs-miR-NC group, the miR-760-3p level was notably diminished, IGF1R protein levels were significantly elevated, and TGF-β1 and p-Smad3/Smad3 levels were dramatically increased in the OSF + EVs-miR-inhi group (all *P* < 0.001) (Figure 7C and 7D). In conclusion, ADSC-EVs improved OSF by inhibiting the TGF-β1/Smad3 pathway by mediating the miR-760-3p/IGF1R axis in mice.

## Discussion

OSF is characterized by abnormal collagen deposition in connective tissues, impacting oral functions and harboring the potential to progress into malignancy [5]. Notably, EVs play a crucial role in the initiation and progression of oral disorders [36]. Moreover, miRNAs are implicated in the regulation of fibrogenesis and carcinogenesis during OSF [37]. EVs, known to encapsulate miRNAs, can modulate the translational profile of recipient cells and influence cellular morphology [38]. In this study, we delved into the mechanistic actions of ADSC-EVs in OSF, with a specific focus on miR-760-3p.

Previous research has established the involvement of ADSCs in the development of fibrosis [39]. Here, we isolated ADSC-EVs and employed an OSF mouse model induced by arecoline feeding, subsequently isolating fibroblasts from these mice (fBMFs). Given the strong correlation between myofibroblast activity in fBMFs and OSF progression [40], these cells served as a relevant model. Co-incubation of fBMFs with ADSC-EVs revealed the internalization of ADSC-EVs by fBMFs. MiRNAs are well-recognized players in OSF development [41], and the observed encapsulation of miR-760-3p in EVs aligns with the accumulating reports on the involvement of EVs in diverse biological processes by transporting miRNA, mRNA, and proteins [42–44]. Our results demonstrated that ADSC-EVs hindered migration and invasion, promoted apoptosis, and reduced the levels of α-SMA, collagen I, and collagen III in fBMFs. Interestingly, silencing miR-760-3p in EVs partially counteracted these effects of ADSC-EVs.

There is robust evidence indicating that OSF-derived myofibroblasts exhibit an enhanced ability to produce collagen, with elevated expression of α-SMA in OSF specimens, linking to the tumor-node-metastasis status in oral carcinomas [45]. In the context of oral diseases, ADSC-EVs have been shown to regulate multiple conditions, preventing the growth and fibrosis of fBMFs via the miR-375/FOXF1 axis [46]. The downregulation of miR-760 in OSF patients and non-cancerous mucosa of individuals with stage IV gastric cancer suggests its potential as a biomarker for precancerous lesions and a molecular therapy target [21, 47]. Collectively, our findings underscore the role of ADSC-EVs in ameliorating the fibrosis of fBMFs, emphasizing the significance of miR-760-3p transport by ADSC-EVs in this context.

miRNAs play a pivotal role in mediating gene expression, orchestrating significant changes in various physiological and pathological processes by binding to target mRNAs [48]. A relevant example is miR-320a, which directly regulates IGF1R in pulmonary fibroblasts [49]. Similarly, our investigation highlights that miR-760-3p targets IGF1R in fBMFs. Notably, the overexpression of IGF1R promoted cell proliferation, migration, and invasion, inhibited apoptosis, and heightened fibrosis of fBMFs. Elevated IGF1 expression in OSF tissues, coupled with the dependence of areca nut-induced fibroblast proliferation on IGF1R, underscores the importance of IGF1R in OSF pathology [50]. Fibroblasts derived from OSF contribute to increased migration and invasion, inducing an epithelial–mesenchymal-like state by upregulating IGF1R in oral cancer cells [51]. Additionally, IGF1R deficiency has been associated with delayed liver fibrosis induced by cholestatic damage [52]. Collectively, ADSC-EVs suppress the fibrosis of fBMFs by inhibiting IGF1R.

Myofibroblasts are key contributors to collagen secretion, culminating in OSF. Identifying targets or pathways that suppress fBMF fibrosis is crucial for intervening in OSF [53, 54]. The pro-fibrotic factor TGF-β, tightly linked to fibrosis and carcinogenesis [55], plays a central role. In OSF, TGF-β1 triggers enhanced collagen production and reduced activity of matrix degradation pathways. ADSC-derived exosomes have been shown to suppress TGF-β1-induced collagen generation in oral mucosal fibroblasts [56]. Studies have highlighted the correlation between cell senescence, fibrosis, and the release of TGF-β1 by senescent epithelial cells, reshaping the microenvironment [57]. CD147, upregulated in fibrotic oral mucosa, correlates with TGF-β1 levels [58]. Repression of TGF-β1 has been linked to the suppression of epithelial–mesenchymal transition, increased apoptosis, and induction of angiogenesis in OSF [59]. Smad3, a key mediator of TGF-β-activated fibrotic processes [60], has been implicated in liver fibrosis mitigation [61]. MiR-497 inhibition hinders myofibroblast transdifferentiation in buccal mucosal fibroblasts through the TGF-β1/Smads pathway, attenuating OSF development [62]. ADSC-exosomes, via the miR-181a-5p/Smad2 axis, exhibit anti-fibrotic effects by modulating myofibroblast proliferation and migration, supporting their potential as a clinical treatment for OSF [63]. Our results demonstrate that ADSC-EVs reduced TGF-β1 and p-Smad3/Smad3 levels in fBMFs, partially counteracted by IGF1R overexpression. This aligns with IGF1R’s facilitation of pulmonary fibrosis through the TGF-β/Smad/STAT cascade [24]. Crucially, in vivo experiments validate the molecular mechanism, showing that ADSC-EVs alleviate OSF by impeding the TGF-β1/Smad3 pathway via the miR-760-3p/IGF1R axis.

## Conclusion

While our study unveils the regulatory role of ADSC-EVs in fBMF fibrosis through the miR-760-3p/IGF1R axis, clinical investigations remain pending due to experimental duration, financial constraints, and the complexity of obtaining clinical samples. Thus, the potential impact of ADSC-EVs on the clinical therapeutic strategy for OSF necessitates further exploration. The study, limited to miR-760-3p/IGF1R, overlooks the roles of other miRNAs in ADSC-EVs and fails to delve into additional downstream target genes and signaling pathways influenced by miR-760-3p. Future efforts will extend validation to the clinical realm, solidifying the status of ADSC-EVs as a promising therapeutic avenue for OSF. Subsequent investigations will broaden the exploration of other miRNAs within ADSC-EVs and unravel additional downstream target genes and related pathways, providing a comprehensive understanding of their regulatory network in fBMF fibrosis. This study establishes that overexpression of IGF1R intensifies colloidal contraction, migration, invasion, and the expression of fibrotic marker proteins in fBMFs, underscoring its pro-fibrotic influence. Further exploration of miR-760-3p’s downstream target reveals its negative modulation of IGF1R expression in fBMFs, implicating it in the myofibroblast activity of fBMFs. This study contributes valuable insights into the intricate molecular mechanisms underlying OSF pathology and offers a foundation for future clinical applications targeting ADSC-EVs in OSF treatment.
